# Inflammatory Response of Human Peripheral Blood Mononuclear Cells and Osteoblasts Incubated With Metallic and Ceramic Submicron Particles

**DOI:** 10.3389/fimmu.2018.00831

**Published:** 2018-04-25

**Authors:** Annett Klinder, Anika Seyfarth, Doris Hansmann, Rainer Bader, Anika Jonitz-Heincke

**Affiliations:** Department of Orthopaedics, Biomechanics and Implant Technology Research Laboratory, Rostock University Medical Center, Rostock, Germany

**Keywords:** wear particles, alumina matrix composite, cobalt-chromium, osteoblasts, peripheral blood mononuclear cells, inflammation

## Abstract

Inflammatory reactions associated with osteolysis and aseptic loosening are the result of wear particles generated at the articulating surfaces of implant components. The aim of the present study was to analyze the biological response of human osteoblasts and peripheral blood mononuclear cells (PBMCs) after exposure to metallic and alumina ceramic particles regarding cellular differentiation, cytokine release, and monocyte migration. Cells were exposed to particles (0.01 and 0.05 mg/ml) from an alumina matrix composite (AMC) ceramic and a CoCr28Mo6 alloy with an average size of 0.5 µm over 48 and 96 h. The expression rates of osteogenic (*Col1A1, ALP*) and pro-osteoclastic (*RANK, Trap5b*) differentiation markers as well as pro-osteolytic mediators (*MMP-1, TIMP-1, IL-6, IL-8, MCP-1*) were determined and soluble protein concentrations of active MMP-1, IL-6, IL-8, and pro-collagen type 1 in cell culture supernatants were evaluated. Additionally, the capacity of particle-treated osteoblasts to attract potentially pro-inflammatory cells to the site of particle exposure was investigated by migration assays using osteoblast-conditioned media. The cellular morphology and metabolism of human osteoblasts and adherent PBMCs were influenced by particle type and concentration. In human osteoblasts, *Col1A1* expression rates and protein production were significantly reduced after exposing cells to the lower concentration of cobalt-chromium (CoCr) and AMC particles. Exposure to AMC particles (0.01 mg/ml) resulted in increased mRNA levels of *RANK* and *Trap5b* in adherent PBMCs. For *MMP-1* gene expression, elevated levels were more prominent after incubation with CoCr compared to AMC particles in osteoblasts, which was not reflected by the protein data. Interleukin (IL)-6 and IL-8 mRNA and protein were induced in both cell types after treatment with AMC particles, whereas exposure to CoCr particles resulted in significantly upregulated IL-6 and IL-8 protein contents in PBMCs only. Exposure of osteoblasts to CoCr particles reduced the chemoattractant potential of osteoblast-conditioned medium. Our results demonstrate distinct effects of AMC and CoCr particles in human osteoblasts and PBMCs. Complex cell and animal models are required to further evaluate the impact of cellular interactions between different cell types during particle exposure.

## Introduction

Steady progress in medicine and biomedical technologies allows the human population to improve their quality of life. Therefore, an increasing number of patients worldwide with degenerative joint diseases underwent total joint arthroplasties (1). However, revision surgery of total joint replacement is still a considerable clinical problem. An analysis of total hip revision data from different arthroplasty registries from around the world (2) showed that the 10-year risk for revision can range from 0.6 up to 66.5% depending on the type of endoprosthetic implants used. The majority of revision surgeries are due to aseptic implant loosening (3), which is associated with a non-specific foreign body response to non-biological influences, such as wear particles or with mechanical problems like stress-shielding (4). The biologic reaction to wear particles consequently leads to the induction of osteolytic processes in the implant surrounding tissue. Occurrence of wear particles is therefore a major factor in long-term failure of endoprosthetic implants (5). A granulomatous inflammatory response is thought to be the initiating event of the osteolytic process in the surrounding bone tissue. This innate immune response is mediated by macrophages, which are specialized to sequester, remove and process foreign body material (6). Furthermore, macrophages can fuse with each other to form multinucleated foreign body giant cells (FBGCs) allowing them to phagocytose wear particles that are too large for a single macrophage. Indeed, histological analysis of periprosthetic tissues adjacent to aseptic loosened implants revealed discrete foreign body granulomas containing macrophages and FBGCs (7). Activated M1 macrophages release pro-inflammatory cytokines that result in osteoclastic differentiation of macrophages and ultimately promote bone resorption (8–10). Increased expression of macrophage-derived chemokines such as interleukin (IL-) 8 and monocyte chemoattractant protein (MCP-) 1 was also demonstrated in periprosthetic tissues in aseptic loosening (11, 12). Consequently, these molecules attract additional macrophages to the site of inflammation.

Number, size, shape, and chemical composition of endoprosthetic wear particles influence phagocytosis by macrophages (13). The amount of abrasive wear varies depending on the articulating implant components such as metal on polyethylene (PE), ceramic on PE, metal on metal, or ceramic on ceramic (14). Simulator tests revealed wear rates of 8.1 mm^3^/10^6^ cycles for CoCr/XPE, 4.7 mm^3^/10^6^ cycles for Al_2_O_3_/XPE, 1 mm^3^/10^6^ cycles for CoCr/CoCr, and 0.052 mm^3^/10^6^ cycles Al_2_O_3_/Al_2_O_3_ (15). Although these wear rates indicated distinct differences between implant components, the ultimate particle amount is unknown since particle numbers depend on size and material composition.

Different tribological pairings might account for the wide variety in failure rates observed by Hughes et al. (2) since different cup and stem combinations do not only generate different amounts of particles but the surrounding tissue might also react differently to the types of particles released from the implant. For example, in cell cultures, viability of macrophages was shown to be higher after phagocytosis of ultra-high molecular weight polyethylene (UHMWPE) and ceramic particles compared to metal particles which promoted apoptosis and necrosis (16–18). Phagocytosis of UHMWPE particles favors osteoclast differentiation (19), while cobalt-chromium (CoCr) particles are associated with necrosis and also trigger adaptive immune response (20).

Apart from macrophages, further cells types, including fibroblasts, lymphocytes, and osteoblasts, are involved in the osteolytic processes leading to implant loosening. Especially, osteoblasts are of importance as they are responsible for new bone formation and counteract bone resorption by osteoclasts. However, in the presence of wear particles, osteoblasts are able to phagocytose the foreign bodies, which subsequently induce the secretion of pro-inflammatory cytokines and chemokines (21, 22). As a consequence, bone forming cells, apart from osteoclasts, are directly involved in bone degradation and progression of aseptic implant loosening. The production of chemokines such as IL-8 and MCP-1 by osteoblasts upon contact with wear particles (23–26) further accelerates the inflammation process. Binding of IL-8 results in activation and recruitment of neutrophils and macrophages (27), while MCP-1 regulates the migration and infiltration of monocytes, natural killer cells, and regulatory T lymphocytes (28).

While the effects of metallic (titanium and CoCr) and UHMWPE particles are relatively well investigated less is known about ceramic particles, especially alumina ceramics, as it was assumed that ceramic materials are bioinert. However, measurement of retrieved ceramic implants revealed higher wear rates than observed in preclinical simulator studies and characteristic “ceramic” wear patterns (29). Due to adverse lubrication conditions or microseparation, the generation of ceramic wear particles can be provoked (30). Consequently, these particles are able to induce a cellular response with a comparable intensity to those caused by metallic and polymeric particles (5). Experiments analyzing the effect of ceramic hydroxyapatite (HA) and β-tricalciumphosphate (TCP) particles on monocytes, polymorphonuclear leukocytes (PMNs), or peripheral blood mononuclear cells (PBMCs) showed increased production of IL-1, IL-6, IL-8, tumor necrosis factor (TNF-) α, macrophage inflammatory protein (MIP-) 1, and matrixmetalloproteinase (MMP-) 8 (31–35) but also reported cytotoxic effects of the particles (36). Calcium phosphate powder furthermore induced MCP-1 and growth-related oncogene (Gro-) α in primary human osteoblasts (26).

Despite similar intensity and outcome molecular mechanisms might differ depending on the material of the particles, as seen for UHMWPE and metal, or depending on the size of the particles. Therefore, the aim of our *in vitro* study was to directly compare the effects of CoCr and alumina matrix composite (AMC) ceramic particles of the same size on human osteoblasts and adherent PBMCs. Both cell types were exposed to submicron particles in the concentration of 0.01 and 0.05 mg/ml to evaluate the biological response of osteoblasts and PBMCs concerning events of bone formation, degradation, and inflammation. Furthermore, we investigated how soluble mediators released by osteoblasts upon contact with particles influenced the migratory potential of isolated monocytes.

## Materials and Methods

### Isolation and Culture of Human Primary Osteoblasts

Human primary osteoblasts (male: *n* = 4, mean age = 64 ± 23; female: *n* = 4, mean age = 76 ± 1) were isolated under sterile conditions from femoral heads of patients undergoing total hip replacement. All samples were collected after patients had signed agreement forms. Approval by the Local Ethics Committee of the University of Rostock (No. AZ: A2010-10) was obtained prior to sample collection.

Trabecular bone was extracted from femoral heads and suspended in phosphate-buffered saline (PBS, Biochrom AG, Berlin, Germany) and washed twice. Human osteoblasts were isolated using the procedure described by Lochner et al. (25).

The obtained osteoblast-like cells were cultured in 25 cm^2^ culture flasks in 8 ml osteogenic medium (Dulbecco’s modified Eagle’s medium (DMEM); Biochrom AG) supplemented with 10% fetal calf serum (FCS, Gibco^®^ Invitrogen, Paisly, UK), 1% penicillin/streptomycin, 1% amphotericin B, 1% hepes buffer, 50 µg/ml ascorbic acid, 10 mM β-glycerophosphate, and 100 nM dexamethasone (all: Sigma-Aldrich, Munich, Germany) and incubated in a humidified atmosphere of 5% CO_2_ at 37°C. Medium change was done every second day and non-adherent cells were aspirated. When a confluence of 100% was reached—as checked by light microscopy—the cells were transferred to 75 cm^2^ culture flasks, further cultured up to a confluence of 100% and stored in 9:1 FCS:DMSO at −150°C for subsequent use.

For further experiments, these cryo-preserved osteoblasts were swiftly de-frosted at 37°C. To remove the conservation medium, the cells were diluted in DMEM, centrifuged at 118 × *g* for 8 min and the cell pellet was resuspended in DMEM containing supplements as described above. Afterward, cells were cultivated in 75 cm^2^ cell culture flasks for seven days at 5% CO_2_ and 37°C in a humidified atmosphere. Thereby, human osteoblasts in passage three were only used for the experiments. A cell number of 1 × 10^4^ (in duplicates) was transferred into a well of a standard 24-well cell culture plate allowing cell adherence over 24 h at 37°C and 5% CO_2_.

### Isolation and Culture of Human Peripheral Blood Mononuclear Cells (PBMCs)

Human PBMCs were isolated from buffy coats from apparently healthy donors, which were provided by the Institute of Transfusion Medicine, Rostock University Medical Center. The provision of buffy coats was made anonymously without indication of gender and age. The procedure was approved by the Local Ethical Committee (No. AZ: A2011-140). Isolation of PBMCs was done by density gradient centrifugation at 320 × *g* and 230 × *g* with Histopaque^®^-1077 (Sigma Aldrich) accordingly to the protocol described in previous work (37). Cells were cultivated in Roswell Park Memorial Institute (RPMI) 1640 medium (Biochrom AG) supplemented with 5% FCS (Gibco^®^ Invitrogen), 1% penicillin/streptomycin, and 1% l-glutamine (both: Sigma-Aldrich) at 37°C and 5% CO_2_. After 7 days in suspension cultures (repellent culture plates provided by Greiner bio one, Frickenhausen, Germany), the cell suspension was centrifuged at 118 × *g* and a cell number of 4 × 10^5^ (in duplicates) was transferred into a well of a standard 24-well cell culture plate allowing cell adherence over 72 h at 37°C and 5% CO_2_. After 72 h, supernatant and non-adherent cells were removed, and the adherent PBMCs were incubated with particles or control medium as described below.

### Particle Characteristics

**Figure 1:**
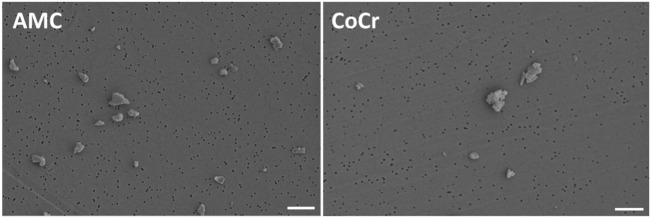
Representative FESEM pictures of AMC and CoCr particles. AMC particles showed a granular, irregular, and angulated appearance (left panel). Particles derived from a CoCr alloy revealed a flake-like to globular (cauliflowers) appearance (right panel). Bar: 1 µm.

Metallic and ceramic abrasive particles were purchased from Continuum Blue (Cardiff, UK). The particles were generated from an AMC ceramic and a cobalt-chromium-molybdenum alloy (CoCr28Mo6). A contamination with endotoxins was excluded after particle production. The mean particle size was 500 nm (manufacturer’s specifications). Particle morphology according to ASTM-F1877-05 was analyzed by field emission scanning microscopy (FESEM, MERLIN VP Compact VP, Carl Zeiss, Oberhausen, Germany). Particles from AMC showed a granular, irregular, and angulated appearance. Particles derived from the CoCr alloy revealed a flake-like to globular (cauliflowers) appearance (Figure 1). The purity of the particles was proven in subsequent studies by EDX. In order to avoid agglomeration, particles were stored in 70% ethanol (EtOH) in a stock solution of 1 mg/ml. The respective particle number per milligram was unknown.

Prior to the cell experiments, particle size in aqueous solution was determined by dynamic light scattering (DLS) measurements (Zetasizer NS, Malvern Instruments, Malvern, UK). The z-Average Diameter (Z_D_) of AMC particles was 1,210 nm and the Polydispersity Index (PdI) was 0.267. The Z_D_ and PdI of CoCr particles were 1,686 nm and 0.221. Although EtOH was used to avoid particle agglomeration, the Z_D_ results indicated agglomerated particles in both particle solutions. The measured PdIs were mid-range values (0.08–0.7) indicating a moderate particle size distribution which was also proven by FESEM (Figure 1).

### Exposure of Cells With Ceramic and Metallic Particles

For subsequent particle dilutions, which were used in the cell experiments, the stock solution was homogenized by ultrawave sonication (UP.H, Hielscher Ultrasonics GmbH, Teltow, Germany) for 2 min each at an amplitude of 80% and a cycle of 0.4. The preparation of the particle dilutions (0.01 and 0.05 mg/ml) was done with the respective cell culture medium (DMEM/RPMI1640) with additives. Previous experiments showed that the applied particle concentrations had bioactive effects (25, 38, 39). Since the material density of the CoCr alloy is 8.3 and 4.37 g/cm^3^ for AMC (29), cells were exposed to approximately double the number of AMC compared to CoCr particles. Additionally, a control solution of DMEM/RPMI1640 and the respective dilution of ethanol (70%) were prepared. Prior to the main experiments the influence of 70% EtOH on cellular viability was excluded by similar results compared to untreated cells. Human osteoblasts and PBMC cultures were treated with particles and EtOH control over a period of 48 and 96 h.

### Metabolic Activity

Viability of human osteoblasts and PBMCs after exposure to particles was determined by the metabolic activity assay WST-1 (Roche, Penzberg, Germany). For the test procedure, the particles were removed and cells were washed with PBS. Afterward, cells were incubated with a defined volume of WST-1/medium reagent (ratio 1:10) at 37°C and 5% CO_2_ for 30 min. Supernatants of the respective culture condition were transferred into 96-well cell culture plates (in duplicates) to determine the absorption at 450 nm (reference wave length: 630 nm) in a microplate reader (Dynex Technologies, Denkendorf, Germany).

### Actin Staining With DAPI Counterstain

Actin staining and DAPI counterstaining was performed to visualize the existence of actin rings in PBMC cultures exposed to particles and EtOH control. After an exposure time of 48 h, the particles were removed and the cells were washed with PBS. All following steps were performed protected from light. The cells were fixated with 4% paraformaldehyde (PFA; pH: 7.0) at room temperature for 10 min. After washing for 30 s, the cell membrane was made permeable with 0.5% Triton-X for 5 min at room temperature. Afterward, the cells were washed with PBS and incubated with 100 nM Acti-stain 488 Fluorescent Phalloidin (Cytoskeleton, Dencer, CO, USA) for 30 min at room temperature. After washing with PBS thrice, the cells were treated with diamidino-2-phenylindole dihydrochloride (DAPI) for 5 min at room temperature. Pictures for actin staining were taken at 500 nm where actin stain fluoresced green. DAPI stain was induced at 400 nm and fluoresced blue. Both pictures were taken from exactly the same spot and layered upon each other with the help of an image processing software (Adobe Photoshop CS6, Adobe Systems Software Ireland Ltd., Dublin, Ireland).

### Medium Conditioning Using AMC and CoCr Particles

Human osteoblasts were seeded in 6-well cell culture plates with a total number of 5 × 10^5^ cells per well in 3 ml full medium (supplemented DMEM as described above) for 24 h at 37°C and 5% CO_2_.

After 24 h of adherence, the medium was replaced by 3 ml DMEM per well containing CoCr or AMC particles with an absolute concentration of 0.01 mg/ml, respectively. The control contained the respective volume equivalent of EtOH only.

After 96 and 168 h of incubation at 37°C and 5% CO_2_, the medium was collected. The volume of 350 µl medium was taken from of each separate well and conditioned media samples from the same treatments were pooled. For protein analyses, 400 µl of pooled, unfiltered medium was stored at −20°C. The remaining pooled medium was filtered to remove particles, cells and other pollutants and frozen at −20°C for subsequent migration assays.

### RNA Isolation and Reverse Transcription

For RNA isolation, the Direct-zol Kit (Zymo Research, Freiburg, Germany) was used as described in the manufacturer’s protocol. Finally, RNA was eluted into a fresh sterile tube using RNase free water. The RNA concentration was measured using the Tecan-Reader Infinite^®^ 200 Pro with RNase free water as blank. The mean ratios of isolated RNA (absorbance at 260/280) were 1.7 (particle-treated) and 2.0 (control) for osteoblasts and 2.1 for particle-treated and control PBMC cultures.

A reverse transcriptase polymerase chain reaction (RT-PCR) was used to transcribe the RNA into cDNA. For preparation of the samples, the High Capacity cDNA Reverse Transcription Kit (Applied Biosystems, Forster City, USA) was used. The mastermix was prepared as described in the manufacturer’s protocol. The specific amount of each RNA sample containing 50 ng RNA was calculated using the results from concentration measurement and added up to 10 µl with RNase free water in PCR tubes. On top, 10 µl of mastermix was added and mixed well. The samples were placed in a thermocycler (Analytik Jena, Jena, Germany). The following RT-PCR protocol was used: 10 min at 25°C, 120 min at 37°C, 15 s at 85°C. Afterward, the samples were diluted in additional 20 µl RNase free water and stored at −20°C.

### Quantitative Real-Time Polymerase Chain Reaction (qRT PCR)

To finally determine the expression rate of genes, the gained cDNA was used to perform a qRT PCR with SybrGreen. For the experiment, the innuMIX qPCR MasterMix SyGreen (Analytik Jena) was used as described in the manufacturer’s protocol. The mastermix was prepared by using 5 µl 2× innuMIX qPCR MasterMix SyGreen, 0.5 µl of forward and reverse primer (12 µM), and 3 µl Aqua dest. For each cDNA sample, 1 µl template cDNA was pipetted onto the bottom of a 96—well Multiply—PCR plate. The used cDNA sequences of osteogenic (*Col1A1, ALP*) and osteoclastic (*RANK, Trap5b*) differentiation marker as well as pro-osteolytic mediators (*IL-6, IL-8, MCP-1, MMP-1, TIMP-1*) are listed in Table 1. To each well, 9 µl mastermix was added. As negative control distilled water instead of cDNA was used as duplicate for each gene. The plate was sealed with adhesive foil and placed in the qTower 2.0 (Analytik Jena).

**Table 1:** cDNA target sequences for qRT PCR.

Primer	Forward (sequence 5′–3′)	Reverse (sequence 5′–3′)	NCBI RefSeq	Product length	Dime formation	Secondary structure
Alkaline phosphatase (ALP)	cattgtgaccaccacgagag	ccatgatcacgtcaatgtcc	NM_000478.5	173	No	Weak/very weak
Collagen 1 (Col1A1)	acgaagacatcccaccaatc	agatcacgtcatcgcacaac	NM_000088.3	129	No	None/very weak
Hypoxanthine-guanine phosphoribosyltrans-ferase (HPRT)	ccctggcgtcgtgattagtg	tcgagcaagacgttcagtcc	NM_000194.2	139	No	None/weak
Interleukin 6 (IL-6)	tggattcaatgaggagacttgcc	ctggcatttgtggttgggtc	NM_000600.3	210	No	None/none
Interleukin 8 (IL-8)	tctgtgtgaaggtgcagttttg	atttctgtgttggcgcagtg	NM_00584.3	144	No	Weak/weak
Matrix metalloproteinase 1 (MMP-1)	agagcagatgtggacatgc	tcccgatgatctcccctgac	NM_001145938.1	131	No	Weak/none
Monocyte chemotactic protein 1 (MCP-1)	ccgagaggctgagactaacc	ggcattgattgcatctggctg	NM_002982.3	157	No	None/weak
Receptor activator of nuclear κ-b (RANK)	agaaaaccaccaaatgaacccc	gccacaagcctcattgatcc	NM_001270949.1	165	No	None/none
Tartrate-resistant acid phosphatase 5b (Trap5b)	gggagatctgtgagccagtg	gtccacatgtccatccaggg	NM_001111034.1	239	No	Weak/none
Tissue inhibitor of metallo-proteinase 1 (TIMP-1)	attgctggaaaactgcaggatg	gtccacaagcaatgagtgcc	NM_003254.2	191	No	Weak/weak

Quantitative real-time PCR analysis was done under the following conditions: an initial activation time of 2 min at 95°C was followed by 40 times of rotation of denaturation of 5 sec at 95°C and annealing/elongation for 25 sec at 60–65°C. The reactions were performed as duplicates. A cycle of threshold (Ct) of 28 was set as limit. The relative expression of each gene compared to the housekeeping gene HPRT was calculated using the equation: ΔCt = Ct_target_−Ct_HPRT_. The relative amount of target mRNA of cells treated with particles and controls was calculated using 2^(−ΔΔCt)^ with ΔΔCt_treatment_ = ΔCt_target_ − ΔCt_control_.

### Determination of Protein Synthesis Rates

Synthesis rates of soluble proteins of pro-collagen type 1 (C1CP; TECOmedical GmbH, Buende, Germany), active MMP1 (Fluorokine E Human Active MMP1; RnDSystems, Minneapolis, MN, USA), IL-6 and IL-8 (both: ThermoFisher Scientific, Waltham, MA, USA), RANKL (Bosterbio, Pleasaton, CA, USA) as well as M-CSF (R&D Systems, Minneapolis, MN, USA) of osteoblasts and PBMCs treated with particles and EtOH control were determined using enzyme-linked immunosorbent assay (ELISA), respectively. Therefore, collected supernatants of particle-treated and control cells (48, 96; 96, 168 h) were used. The ELISAs were used according to the manufacturer’s protocol. Fluorescence/Absorbance measurement was performed utilizing a Tecan-Reader Infinite^®^ 200 Pro (Maennedorf, Switzerland) at 320 nm (excitation) and 405 nm (emission) for MMP1, 405 nm (reference wave length: 630 nm) for C1CP, IL6, and IL8 as well as 450 nm for M-CSF (reference wave length: 550 nm) and RANKL. Using a standard curve, the respective protein content was calculated. Afterward, all data were normalized with the overall protein content measured by the Qubit Protein Assay Kit and Qubit 1.0 (ThermoFisher Scientific) following the manufacturer’s protocol.

### Migration Assay

For analysis of monocyte migration, human monocytes were isolated from previously isolated PBMCs using monocyte isolation kit with Dynabeads^®^ (Thermo Fisher Scientific) according to the manufacture’s protocol. The resulting supernatant containing the desired monocytes was centrifuged at 120 × *g* for 8 min at room temperature. The cell pellet was resuspended in a defined amount of RPMI 1640 and counted.

For the migration experiment bead-isolated monocytes were seeded with 300,000 cells per insert with 250 µl RPMI 1640 supplemented with 2% FCS, 1% penicillin/streptomycin, and 2% l-glutamine in 24-well cell culture inserts. Subsequently, 250 µl supplemented RPMI 1640 plus 250 µl osteoblast-conditioned DMEM were added to the wells below (in duplicate). The following mediators were used as positive controls in the bottom wells: human sRANKL (50 ng/ml in supplemented RPMI 1640/DMEM [1:1 v/v], Peprotech Inc., Rocky Hill, NJ, USA), human M-CSF (25 ng/ml in supplemented RPMI 1640/DMEM [1:1 v/v], Peprotech Inc., Rocky Hill, NJ, USA) or the combination of human sRANKL and +M-CSF (50 and 25 ng/ml in supplemented RPMI 1640/DMEM [1:1 v/v], respective). Supplemented RPMI 1640/DMEM (1:1 v/v) without additional growth factors was used as a negative control. The cells were incubated for 18 h at 37°C and 5% CO_2_.

In order to determine the number of migrated cells, suspension cells in the supernatants from the inserts and the bottom wells were collected and transferred to separate tubes. Adherent cells were harvested by trypsinization from inside of the insert, the lower surface of the insert, and the bottom of the well. After centrifugation at 120 × *g* for 8 min at room temperature, all samples were counted using a Thoma hemacytometer. The single cell counts were combined as follows: (a) cells in insert—suspension cells in the supernatant from the insert plus adherent cells from inside of the insert and (b) cells in well—adherent cells from the lower surface of the insert plus suspension cells in the supernatant of the bottom well and adherent cells from the bottom of the well.

### Statistical Analysis and Data Illustration

Data are shown as box plots. Boxes depict interquartile ranges, horizontal lines within boxes depict medians and whiskers depict maximum and minimum values. For analysis, cell cultures of osteoblasts and PBMCs were used in duplicates with a minimum of four independent donors. If the obtained data were not distributed homogeneously, statistical significances were calculated with non-parametric tests using SPSS 20 (IBM Deutschland, Ehningen, Germany). Statistical tests were performed as indicated in the results section and the figure legends. Significances were set to a *p*-value that is equal to or less than 0.05.

## Results

### Effect of Particles on Cellular Morphology and Viability

**Figure 2:**
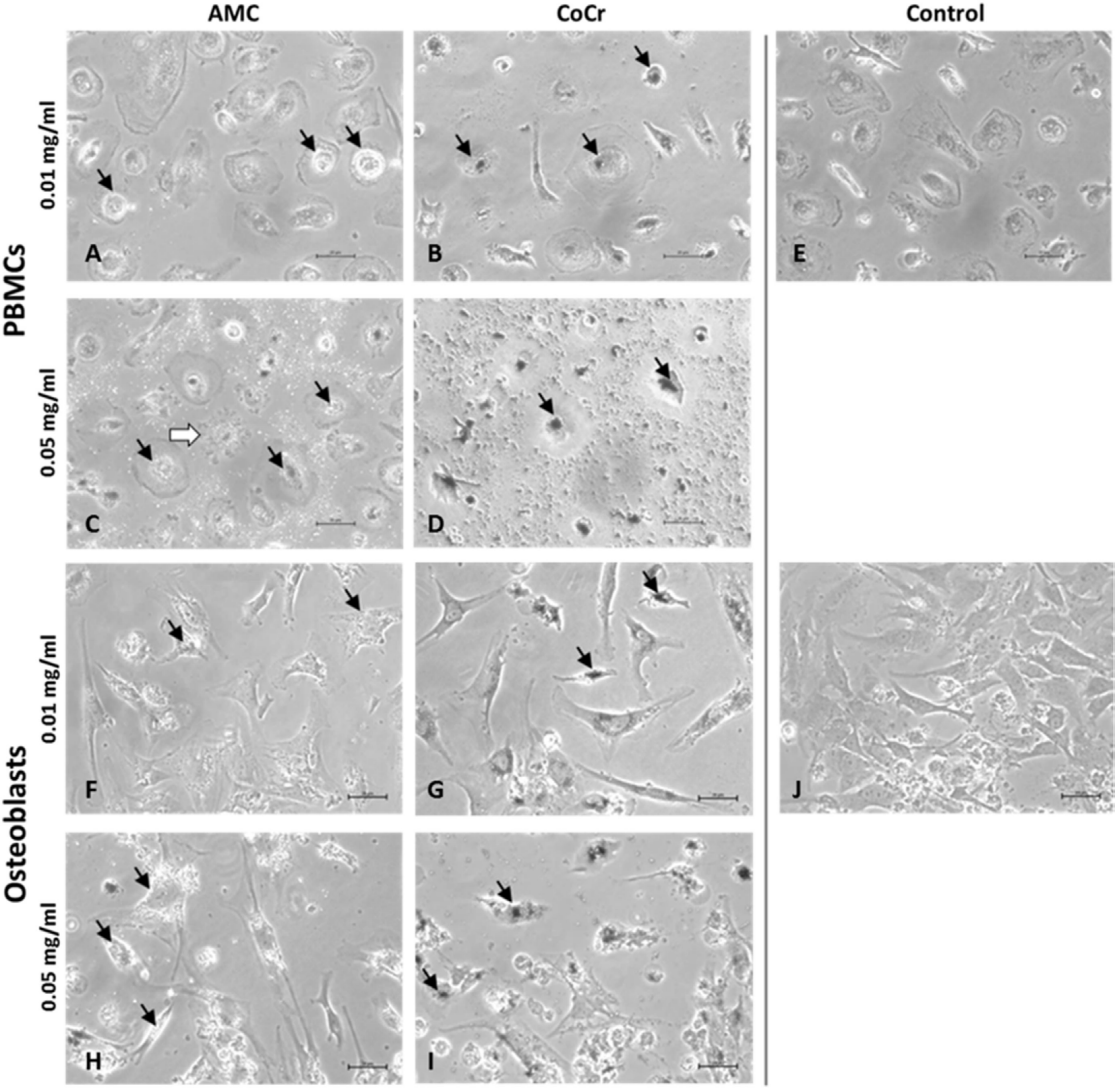
Representative pictures of human PBMCs **(A–E)** and osteoblasts **(F–J)** after exposure to AMC and CoCr particles. Cells were treated with the respective particle type using different concentrations over a period of 48 h. Particle accumulations are highlighted with black arrows. Light microscopy revealed either particle- or concentration-dependent effect on cell density and morphology. Bar: 20 µm.

Evaluation of cells by light microscopy revealed distinct morphological changes in human osteoblasts and adherent PBMCs depending on the type of particle and the applied concentration (Figure 2). AMC particles seemed to be phagocytized by human PBMCs as well as osteoblasts (Figures 2A,C,F,H). At the higher concentration, AMC particles lined the outside of the osteoblasts (Figure 2H). Phagocytosis of AMC resulted in less change in the morphology of the incubated cells, however, after incubation of adherent PBMCs with 0.05 mg/ml AMC particles cells with ruffled borders and finger-like shapes at the cell membrane occurred in these cell cultures (Figure 2C, white arrow).

Changes in cell morphology were more prominent after incubation with CoCr particles. The accumulation of phagocytized CoCr particles was observed in adherent PBMCs (Figure 2B,D black arrows). While adherent PBMCs resembled mainly a phenotype of M1 macrophages (Figure 2E) incubation with 0.05 mg/ml CoCr particles resulted in enlarged, flat cells with barely visible borders which were difficult to distinguish in phase contrast (Figure 2D). Human osteoblasts lost their elongated, spindle-like appearance, which is usually observed in cell culture (Figure 2J), especially at the higher concentration of 0.05 mg/ml (Figure 2I). The osteoblasts incubated for 48 h with 0.05 mg/ml CoCr particles appeared rounder, shorter, and also fragmented with an accumulation of phagocytized CoCr particles clearly visible inside the cells (Figure 2I, black arrows).

**Figure 3:**
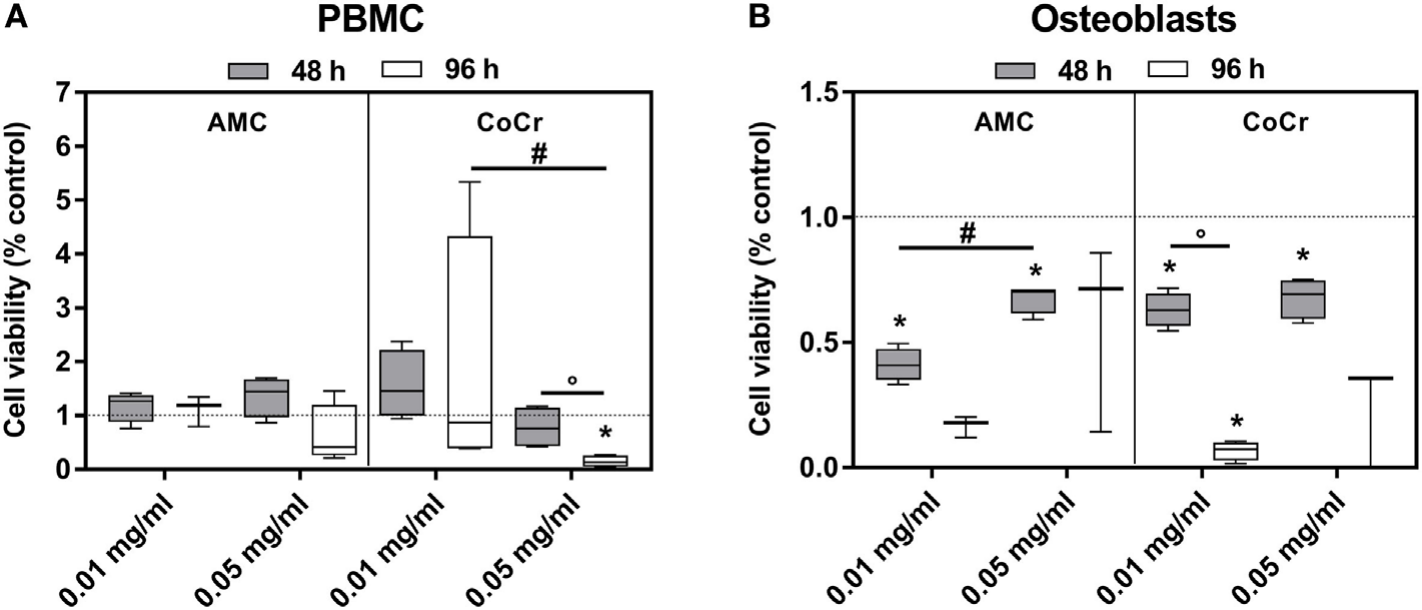
Cell viability of human PBMCs **(A)** and osteoblasts **(B)** after exposure to AMC and CoCr particles. Cells treated with the respective particle were analyzed using the WST-1 assay. Data are shown as box plots (*n* ≥ 3). Significances between groups were calculated with the Mann–Whitney *U*-test (*p < 0.05, treated vs. untreated; **^#^***p* < 0.05, 0.01 vs. 0.05 mg/ml; °*p* < 0.05, 48 vs. 96 h).

The observations from the light microscopy were supported by the results of the viability assay (Figure 3). The incubation of PBMCs with AMC particles had barely any influence on the cell activity as measured by WST-1 assay (Figure 3A), while in osteoblasts exposure to AMC particles, especially to the lower concentration, significantly inhibited cell growth in a time-dependent manner compared to the control (Figure 3B). It is not entirely comprehensible why the lower concentration should have a more pronounced effect. As the WST-1 assay does not measure growth directly, the higher values after exposure to 0.05 mg/ml AMC particles might not indicate increased growth compared to 0.01 mg/ml but higher cell activity, e.g., as response to increased oxidative stress.

Exposure to CoCr particles resulted in pronounced cytotoxic effects in PBMCs and in osteoblasts. While PBMCs were still able to tolerate the lower concentration of 0.01 mg/ml CoCr particles 0.05 mg/ml nearly completely reduced the cellular activity of PBMCs after 96 h (Figure 3A). Osteoblasts were more sensitive than PBMCs. Both concentrations significantly decreased cell viability after 48 h and suppressed the growth/cellular activity after 96 h (Figure 3B).

### Effect of Particles on Differentiation Markers

**Figure 4:**
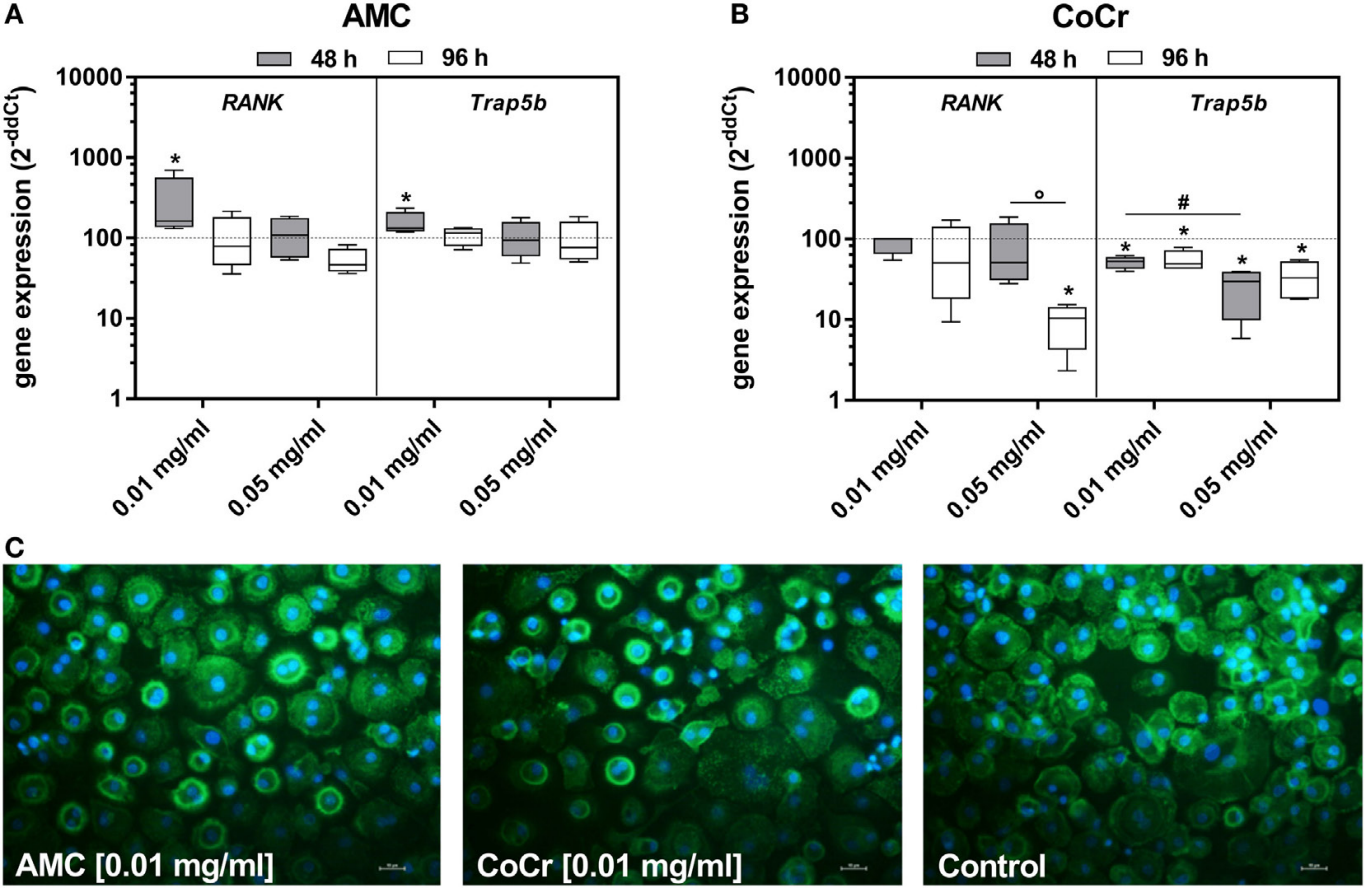
Differentiation capacity of human PBMC cultures after exposure to AMC and CoCr particles in different concentrations. Cells were analyzed for gene expression levels of pro-osteoclastic differentiation marker by qRT-PCR **(A,B)**. Data are shown as box plots (*n* = 4). Significances between groups were calculated with the Mann–Whitney *U*-test (*p < 0.05, treated vs. untreated; **^#^***p* < 0.05, 0.01 vs. 0.05 mg/ml; °*p* < 0.05, 48 vs. 96 h). **(C)** Representative pictures of actin (green fluorescence) and cell nuclei (blue fluorescence) in PBMC cultures after 48 h of incubation with AMC and CoCr particles. Exposure to particles revealed an enhanced cell number with actin rings as well as formation of membranous ruffled border compared to untreated controls. Bar: 20 µm.

In adherent PBMCs exposure to CoCr particles suppressed the gene expression of osteoclastic differentiation markers *RANK* and *Trap5b* in a concentration-dependent manner (Figure 4B). Contrarily, incubation of PBMCs with 0.01 mg/ml AMC particles significantly induced the gene expression of *RANK* and *Trap5b* after 48 h (Figure 4A). Additionally, an increased occurrence of actin rings in adherent PBMCs after incubation with AMC particles was observed (Figure 4C).

**Figure 5:**
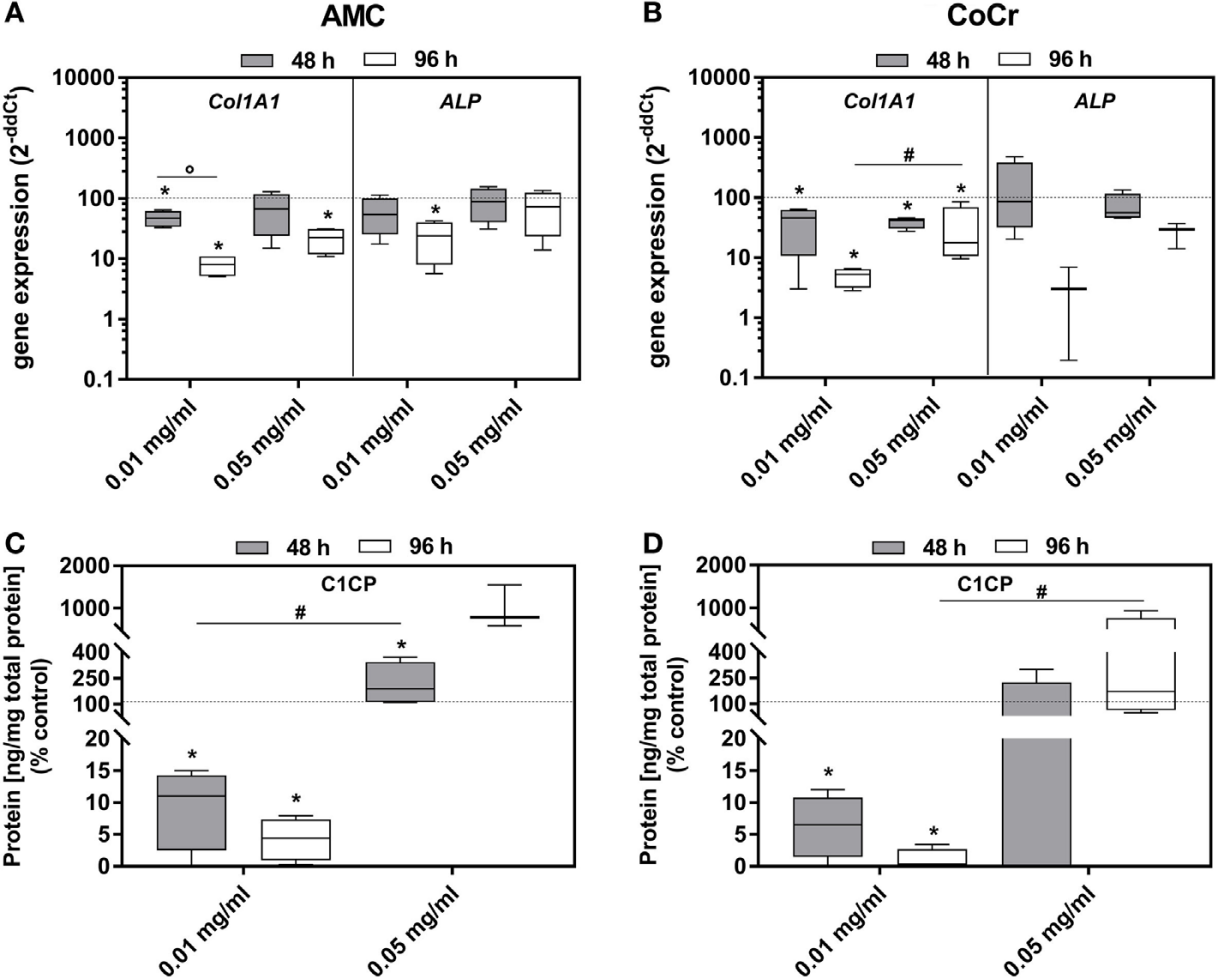
Differentiation capacity of human osteoblasts after exposure to different concentrations of AMC and CoCr particles. Cells were treated with the respective particle to evaluate gene expression levels of osteogenic differentiation marker by qRT-PCR **(A,B)**. **(C,D)** Cell culture supernatants were used to evaluate the concentration of pro-collagen type 1 (C1CP per total protein content). Data are shown as box plots (*n* ≥ 3). Significances between groups were calculated with the Mann–Whitney *U*-test (*p < 0.05, treated vs. untreated; **^#^***p* < 0.05, 0.01 vs. 0.05 mg/ml; °*p* < 0.05, 48 vs. 96 h).

Incubation of osteoblasts with the particles influenced the gene expression of *Col1A1* and *ALP*. Both, AMC and CoCr wear particles, significantly reduced the gene expression of these markers associated with osteoblast differentiation (Figures 5A,B). The effect was more pronounced after 96 h of incubation, even if the difference between the time points was only significant for *Col1A1* expression after incubation with AMC particles (*p* = 0.029). The lower concentration of AMC and CoCr particles of 0.01 mg/ml seemed more effective in reducing expression of *Col1A1* (96 h: *p* = 0.029) and *ALP* compared to 0.05 mg/ml. The reduction of *Col1A1* gene expression rates after exposure to 0.01 mg/ml of both types of particles was confirmed by significantly decreased protein amounts of pro-collagen type 1 (all: *p* = 0.029) after these treatments. Surprisingly, the higher particle concentration induced the production of pro-collagen type 1 protein despite reduced gene expression rates compared to the untreated control (Figure 5C,D). However, the increase was only significant for AMC particles after 48 h (*p* = 0.029; Figure 5C).

### Induction of Pro-Osteolytic Mediators

**Figure 6:**
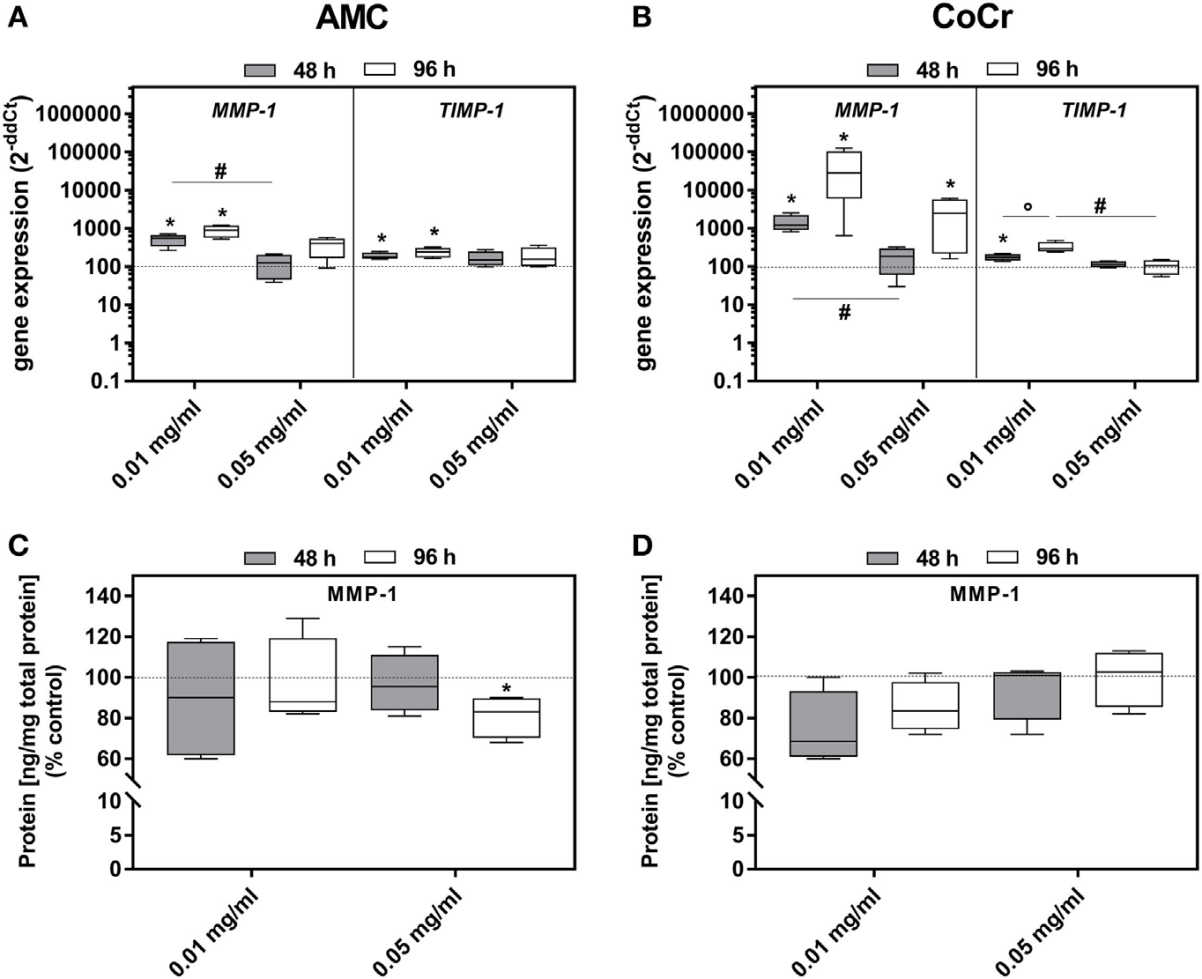
MMP-1 and TIMP-1 expression rates in human osteoblasts following exposure to AMC and CoCr particles. Gene expression levels of *MMP-1* and *TIMP-1* by qRT-PCR **(A,B)** were determined in cells treated with the respective particle type. **(C,D)** Cell culture supernatants were used to evaluate the concentration of activated MMP-1 protein (per total protein content). Data are shown as median and minimum/maximum values (*n* = 4). Significances between groups were calculated with the Mann–Whitney *U*-test (*p < 0.05, treated vs. untreated; **^#^***p* < 0.05, 0.01 vs. 0.05 mg/ml).

Incubation with AMC or CoCr significantly induced the gene expression of *MMP-1* as well as *TIMP-1* in osteoblasts (Figures 6A,B). Similar to the expression of osteoblastic differentiation markers the lower concentration was more effective. For *MMP-1* gene expression, elevated levels were more prominent after incubation with CoCr compared to AMC particles. The elevated gene expression of *MMP-1* in osteoblasts was not reflected by increased protein amounts of active MMP-1 (Figures 6C,D). The higher concentration of 0.05 mg/ml AMC particles even reduced the amount of active MMP-1 protein significantly. In PBMC cultures, there were no significant changes in gene expression rate of *TIMP-1*. *MMP-1* mRNA and active MMP-1 were not detected in PBMCs.

**Figure 7:**
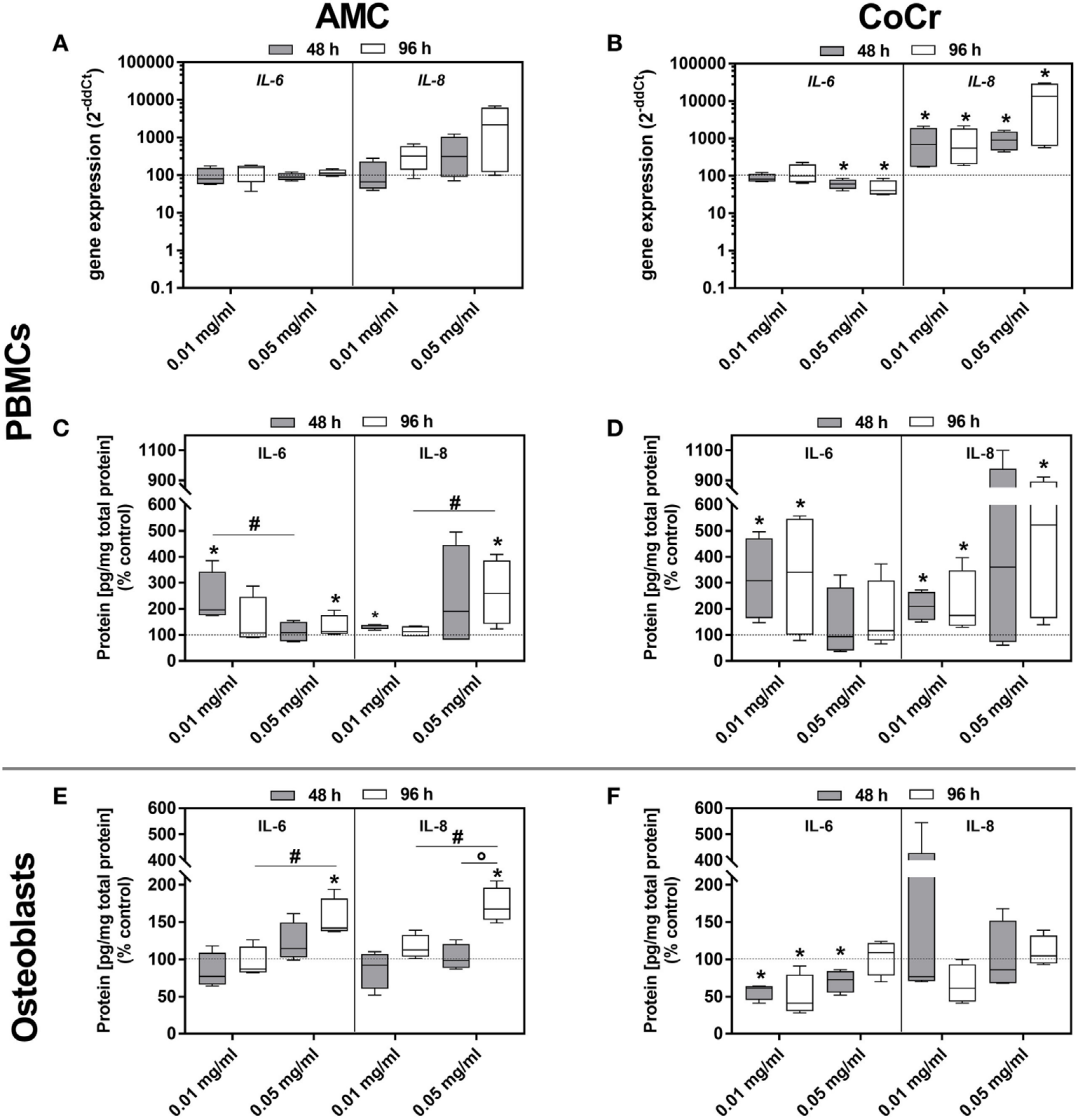
IL-6 and IL-8 expression rates in human PBMC cultures and osteoblasts following exposure to AMC and CoCr particles. PBMCs were treated with the respective particle to evaluate gene expression levels of *IL-6* and *IL-8* by qRT-PCR **(A,B)**. **(C–F)** Cell culture supernatants of particle-exposed PBMCs and osteoblasts were used to evaluate the concentration of soluble IL-6 and IL-8 protein (per total protein content). Data are shown as median and minimum/maximum values (*n* = 4). Significances between groups were calculated with the Mann–Whitney *U*-test (*p < 0.05, treated vs. untreated; **^#^***p* < 0.05, 0.01 vs. 0.05 mg/ml).

The picture was more complex for the inflammation markers. In adherent PBMCs *IL-8* expression was upregulated in a dose-dependent manner by AMC and CoCr particles. The effect was more pronounced after incubation with CoCr particles. The protein data for IL-8 corresponded with the dose-dependent increase in gene expression observed in PBMCs exposed to AMC and CoCr particles (Figures 7C,D). Gene expression of *IL-6* increased in PBMCs after exposure to AMC particles, while incubation with a high concentration of CoCr particles resulted in a significant decrease in *IL-6* expression (Figures 7A,B). Despite downregulated gene expression for *IL-6* in PBMCs, elevated levels of IL-6 protein in the supernatant of adherent PBMCs were observed in the presence of particles (Figures 7B,D).

Although gene expressions of *IL-6* and *IL-8* after incubation with AMC and CoCr particles were not induced in human osteoblasts, a concentration- and time-dependent increase of IL-6 and IL-8 protein amounts was detected after exposure to AMC particles (Figure 7E). Contrarily, IL-6 protein was significantly downregulated after exposure to CoCr particles (Figure 7F).

Only the exposure to the higher AMC particle concentration resulted in significantly downregulated *MCP-1* gene expression after 96 h in PBMCs. Additionally, treatment with CoCr particles also tended to decrease *MCP-1* mRNA levels. In osteoblasts, the lower concentrations of AMC particles reduced gene expression of *MCP-1*. Exposure to CoCr particles did not induce *MCP-1* in osteoblasts (data not shown).

### Particle-Induced Migration of Monocytes

Osteoblast-conditioned media were used to induce migration of human monocytes. In order to generate these conditioned media, human osteoblasts were exposed to AMC and CoCr particles for 96 and 168 h, medium containing a volume equivalent of EtOH (solvent used to store CoCr and AMC particles) was used as a negative control. The osteoblast-conditioned media were tested for contents of M-CSF, IL-6, IL-8, and RANKL proteins. Protein content of the different mediators in the pooled samples, which were used for the migration assays, is shown in Table 2. RANKL protein contents were not detected in the osteoblast-conditioned media in any group.

**Table 2:** Protein concentration in conditioned media.

Mediator	AMC		CoCr		Control (EtOH)	
	96 h	168 h	96 h	168 h	96 h	168 h
M-CSF (pg/μg)	0.86	1.61	0.64	1.08	1.34	2.96
IL-6 (pg/μg)	0.67	0.76	0.47	0.43	0.90	1.13
IL-8 (pg/μg)	1.10	1.17	0.94	1.11	0.88	1.03

*Osteoblasts (*n* = 4) were exposed to AMC and CoCr (concentration: 0.01 mg/ml). Cell culture supernatants of all donors were pooled to evaluate the mean protein concentrations (in relation to the total protein content)*.

**Figure 8:**
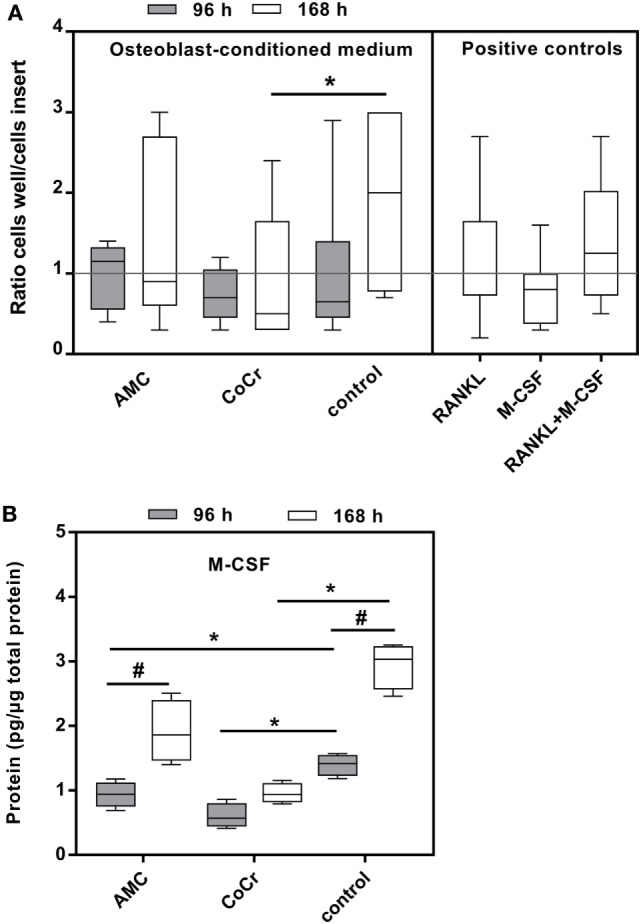
Osteoblast-conditioned medium: influence on migration of human monocytes **(A)** and M-CSF protein content **(B)**. **(A)** Cells were treated with 96 and 168 h osteoblast-conditioned medium and growth factor controls (positive controls) for 18 h. Cells in insert and well were counted and ratios calculated. Data are shown as box plots of ratios (*n* = 6) normalized to the negative control. Significances between groups were calculated with Friedman test with Dunn’s post-test (*p < 0.05). **(B)** Cell culture supernatants were used to evaluate the concentration of M-CSF (per total protein content). Data are shown as box plots (n = 4). Significances between groups were calculated with the Mann–Whitney *U-*test (*p < 0.05, treated vs. untreated; **^#^***p* < 0.05, 96 vs. 168 h).

Incubation of isolated monocytes with osteoblast-conditioned media resulted in the successful migration of a certain proportion of monocytes through the insert membrane after 18 h. Migration values are depicted as ratios of cells migrated into the well to cells in the insert, normalized to the medium control (negative control), i.e., the higher the value the more cells migrated into the well and values higher than “1” indicated higher migration compared to medium control. Control medium conditioned by osteoblasts for the duration of 168 h and the positive control containing both, synthetic M-CSF and RANKL, seemed to favor migration, however, due to high interindividual variation, probably due to the monocyte donors, these changes did not reach significance (Figure 8A). Surprisingly, medium conditioned by osteoblasts after exposure to CoCr particles for 168 h significantly suppressed migration compared to osteoblast-conditioned control medium (*p* = 0.0283, Friedman test with Dunn’s multiple comparison as post-test). AMC particle exposure showed no influence. Interestingly, M-CSF protein concentration was also significantly lower in the medium conditioned by osteoblasts after exposure to CoCr particles (Figure 8B), and there was a significant correlation between the median of migration ratios and the M-CSF protein concentration in the conditioned media (*r* = 0.823, *p* = 0.044, Pearson correlation). However, the positive control of synthetic M-CSF was not effective. There were no cytotoxic effects of the osteoblast-conditioned media or the positive controls on the monocytes (data not shown).

## Discussion

The biological response of cells to abrasive wear particles is triggered by particle number, size and morphology. Due to the biological inertness of polymers and ceramics, these materials have no ability to corrosion. Therefore, the chemical composition of wear particles has a significant influence of the immunological response of cells. Non-metallic wear debris stimulates primarily the innate immune response, whereas metal materials as particles and ions are able to stimulate both, innate and adaptive immune reactions (40).

In case of aseptic implant loosening, osteolysis and bone loss are primarily mediated by the activity of macrophages which phagocytize particles. Apart from macrophages, bone forming cells like osteoblasts were also shown to undertake particle phagocytosis (21, 22). In our present *in vitro* study, we investigated the effects of particles from a cobalt chromium molybdenum alloy and AMC ceramic. Although both particle types are characterized by equal average size of 500 nm, we determined larger particle agglomerates by DLS measurements and FESEM, especially for CoCr particles. However, data from literature revealed that the verified particle size, especially for ceramic particles, is of clinical relevance as it elicits an immunological response. For example, Yagil-Kemer et al. showed a high induction of relevant pro-inflammatory cytokines in human monocytes after exposure of cells to ceramic particles with 500 nm in size (41). Additionally, Lohmann et al. showed the incorporation of ceramic particles in osteosarcoma cells (MG63) with a size of approximately 1 µm (22). Laquerriere et al. showed that only HA particles that were phagocytized were able to induce gene expression and production of IL-6 and TNF-α (31). In contrast, non-phagocytable particles regulated MMP-2, MMP-9, and TIMPs (32). These reports suggest that particle size is critical for studying particle incorporation into human PBMCs and osteoblasts regarding cellular response. Agglomeration products as observed after DLS measurements and FESEM may elicit differing results compared to particles of 500 nm.

### Effects of Ceramic and Metallic Particles on Cellular Morphology and Differentiation

Both, human osteoblasts and PBMCs showed aggregation of AMC and CoCr particles on the cellular membrane. While the direct phagocytosis of particles was not investigated, light microscopical evaluation suggested an accumulation of particles inside the cells. The assumption, that both cell types phagocytized the particles is in accordance with previous *in vitro* studies (21, 22, 41).

Interestingly, we found an inverse particle concentration-dependent effect on osteoblastic and osteoclastic differentiation markers. A possible reason for the reduced effect of the higher particle concentration may be that high concentrations favor the agglomeration of particles in suspension and thus preventing these larger particle agglomerates from being phagocytized.

In contrast to AMC particles, CoCr particles inhibited the induction of pro-osteoclastic differentiation markers *RANK* and *Trap5b* as well the formation of actin rings in PBMCs. The reduced expression levels of RANK and Trap5b are in accordance with our previous study, where exposure to CoCr ions also significantly reduced the pro-osteoclastic differentiation capacity of adherent PBMCs (37). This observation was also associated with a changed cellular appearance of adherent PBMCs, e.g., cells were smaller in diameter and did not show the typical cell sprawling at the lower CoCr particle concentration and were barely visible at the higher concentration. Similarly, Scharf et al. reported a transition from healthy to degenerative cell appearance in macrophages in periprosthetic tissue, which was induced by the greater cytotoxicity of CoCr due to its corrosive behavior (42). The intracellular depletion of particles and release of Co and Cr ions furthermore results in an increased toxic environment for cells (42). The multitude of CoCr particles (0.05 mg/ml) observed outside the PBMC cultures could either suggest that macrophages were completely saturated with particles or that necrosis of macrophages occurred due to cytotoxic effects of the metals and particles were re-released into the medium. Indeed, the results from the WST-1 assay suggest severe cytotoxic effects of the higher particle concentration on osteoblasts as well as on adherent PBMCs.

The reduced gene expression of osteogenic differentiation marker ALP at the lower particle concentration in response to both, ceramic and metallic particles, is in accordance to results of Lohmann et al. (21, 22). Effects on *Col1A1* synthesis rates were comparable between both particle types suggesting a primary effect of particles. Indeed, when comparing the results of *Col1A1* and *ALP* expression after CoCr particle to previously tested ionic exposure, treatment with Co and Cr ions evoked a non-toxic effect (37). However, reduction in osteogenic differentiation is directly associated with cytotoxic reactions like swollen mitochondria and formation of a ruffled border of the cell membrane (21, 22). In contrast to the lower particle concentration, exposure to the higher ones resulted in enhanced pro-collagen type 1 amounts. As mentioned above, the higher particle concentration favors particle agglomeration which could be recognized as a rough surface by osteoblasts (21) resulting in enhanced collagen type 1 production.

### Induction of Inflammatory Processes in Response to Metallic and Ceramic Particles

As it was described by Lohmann et al., exposure of osteoblastic cells with ceramic and metallic wear particles is associated with a higher prostaglandin E2 (PGE2) expression rate (21, 22) which could further induce high levels of *MMP1* mRNA (43). Indeed, we were able to demonstrate a direct induction of *MMP-1* and *TIMP-1* mRNA in human osteoblasts after application of AMC and CoCr particles; however, the active form of MMP-1 protein was not affected by particles. In PBMC cultures, *MMP-1* mRNA and active MMP-1 protein were not detectable and *TIMP-1* gene expression rates were not influenced. These results were unexpected since it was previously reported that particle exposure is associated with high MMP (e.g., MMP-1, MMP-2, MMP-3, MMP-9, and MMP-13) expression rates (5). Furthermore, our previous works indicated relevant MMP-1 expression levels in macrophage and PBMC cultures (37, 39). However, due to the use of isolated single cell cultures in our *in vitro* experiments, signals important for the production, activation, or release of MMP-1 in osteoblasts in form of specific cytokines and chemokines might not be present. In further experiments, this aspect should be investigated using more complex cell models to evaluate the impact of cellular interactions during particle exposure. Moreover, other relevant MMPs and TIMPs might be worth testing to obtain a more specific cellular response to AMC and CoCr particles as it was already shown previously by us that release of MMP-3, MMP-8, and MMP-10 in macrophages were affected (39). Regarding the induction of IL-6 and IL-8 expression rates, we demonstrated a higher impact of AMC particles on the release of soluble IL-6 and IL-8 proteins compared to particles from CoCr after 96 h in human osteoblasts. In PBMCs, on the other hand, AMC as well as CoCr particles clearly upregulated expression levels of IL-6 and IL-8 mRNA and protein secretion of both interleukins. One explanation for these differing effects could be again the corrosion behavior of CoCr, and therefore, the release of ions which could influence the cellular response. Samelko et al. demonstrated that cobalt-alloy particles induced inflammation signaling in macrophages *via* danger-associated molecular patterns (DAMPs) and NLRP3 inflammasome (44), which was already identified as a critical mediator in wear particle induced osteolysis (45). The authors showed that the process is independent from TLR4 activation and suggest that signals released from the lysosomes after phagocytosis of cobalt-alloy particles such as metal ions, reactive oxygen species or Cathepsin B activate the DAMP pathway in macrophages (44). Indeed, in a previous study, we have shown that exposure to CoCr ions upregulated *IL-8* mRNA expression in adherent PBMC cultures (37). We also detected initially higher expression rates of *IL-6* and *IL-8* in human osteoblasts after exposure to metal ions (37). Hence, this supports the assumption that the ionic form plays an important role in osteolysis by stimulating the production of cytokines and chemokines in osteoblasts (46), while the toxicity of CoCr particles is associated with reduced mRNA expression rates of certain mediators as well as osteogenic differentiation markers.

Our data confirm the assumption that the inflammatory response is firstly material-dependent and secondly cell-type specific. Here, particles derived from AMC had a greater impact on the release of cytokines by human osteoblasts and the gene expression of osteoclastic differentiation markers *RANK* and *Trap5b*. To this end, we are not able to speculate whether the response of osteoblasts and PBMCs to AMC particles could result in osteolysis. Since our results showed enhanced protein levels of IL-6 and pro-collagen type 1 after exposure to the higher concentration of AMC particles, it can also be assumed that the cellular reactions are natural effects on bone remodeling processes. High levels of IL-6 protein trigger the release of RANKL by osteoblasts and synovial cells which in turn activate the differentiation of osteoclasts. In dependency to their differentiation stage, IL-6 can also influence the maturation of pre-osteoblasts into osteoblasts. Thus, IL-6 promotes osteoblastic differentiation (47). Therefore, IL-6 seems to be an important mediator in bone remodeling processes.

Since it was shown that osteoblasts reacted with higher PGE2 expression rates after particle exposure (21, 22), it would be interesting to analyze the secretion of PGE2, a known regulator of bone turn-over (5), in response to both particle types. In this context, it will also be interesting to investigate whether the upregulation of PGE2 could stimulate osteoclastic bone resorption (46, 48) in further co-culture experiments, especially by using of AMC particles because of their ability for the inducing of pre-osteoclastic differentiation markers.

### Influence of Osteoblast-Conditioned Medium

Osteoblasts are known to produce substances involved the chemotaxis of monocytes in response to submicron particles (49). Surprisingly, in our *in vitro* assay simulating chemotaxis in response to osteoblast-conditioned medium the exposure to CoCr particles significantly reduced the chemoattractant potential of the osteoblast-conditioned media. The results are nevertheless supported by the data of protein secretion in the collected supernatants since IL-8 was not affected by CoCr exposure, IL-6 secretion was reduced and RANKL was not detected in the osteoblast-conditioned media. The gene expression of *MCP-1* was also significantly reduced in osteoblasts after treatment with CoCr particles (data not shown). The lack of these chemokines would account for the suppression of monocyte migration. Furthermore, M-CSF which correlated with the chemotactic potential of the osteoblast-conditioned media in our experiment was also significantly reduced. We cannot exclude that different concentrations or sizes of particles would elicit a different cellular response.

In summary, we demonstrated differing effects of AMC and CoCr particles on the differentiation and cytokine production in human osteoblasts and PBMCs. Regarding the effects of particles on adherent PBMCs, we found distinct differences in the release of pro-inflammatory cytokines and our results indicated a higher pro-inflammatory potential by CoCr particles as shown by enhanced IL-6 and IL-8 protein levels. However, the potential of AMC particles for inducing cytokines involved in pro-osteoclastic differentiation and inhibiting osteoblastic differentiation at lower particle loadings could indicate a risk of bone resorption processes which needs to be examined in future studies.

A direct comparison of biologic reaction between particle types is limited because of unknown particle number of the used materials. Moreover, with respect to the specific material density, we have exposed cells with approximately double the number of ceramic particles compared to CoCr particles. This is clearly opposite to the *in vivo* situation where the wear-resistant ceramic materials produce lower amounts of wear debris compared to metals resulting in reduced adverse biological reactions and osteolysis (5).

In conclusion, ceramic particles induce a lower immune response compared to CoCr particles but might elicit osteoclastic differentiation *in situ*. Therefore, further co-culture experiments and animal studies should be carried out in order to analyze the biological activity of different wear particles and the pathways triggered *in vivo*.

## Ethics Statement

Within this study, human primary osteoblasts and PBMC cultures were used for the experiments. This study was carried out in accordance with the recommendations of the Local Ethical Committee of the University of Rostock with written informed consent from all subjects. All subjects gave written informed consent in accordance with the Declaration of Helsinki. The protocol was approved by the Local Ethical Committee of the University of Rostock.

## Author Contributions

AK, AS, DH, and AJ-H performed the experiments. Data were analyzed by AK, AS, and AJ-H. The manuscript was written by AK, AS and AJ-H and revised by RB. AJ-H and RB designed the study and applied for the funding.

## Conflict of Interest Statement

The authors declare that the research was conducted in the absence of any commercial or financial relationships that could be construed as a potential conflict of interest.
